# Cytotoxicity of trifluridine correlates with the thymidine kinase 1 expression level

**DOI:** 10.1038/s41598-019-44399-6

**Published:** 2019-05-28

**Authors:** Yuki Kataoka, Makoto Iimori, Shinichiro Niimi, Hiroshi Tsukihara, Takeshi Wakasa, Hiroshi Saeki, Eiji Oki, Yoshihiko Maehara, Hiroyuki Kitao

**Affiliations:** 1 Department of Molecular Cancer Biology, Graduate School of Pharmaceutical Sciences, Kyushu University, 3-1-1 Maidashi, Higashi-ku, Fukuoka 812-8582 Japan; 2 Drug Discovery & Development I Laboratory, Taiho Pharmaceutical Co. Ltd., 3, Okubo, Tsukuba, Ibaraki 300-2611 Japan; 3 Innovative Anticancer Strategy for Therapeutics and Diagnosis Group, Innovative Center for Medical Redox Navigation, Kyushu University, 3-1-1 Maidashi, Higashi-ku, Fukuoka 812-8582 Japan; 4 Translational Research Laboratory, Taiho Pharmaceutical Co., Ltd., 224-2 Ebisuno Hiraishi, Kawauchi-Cho, Tokushima 771-0194 Japan; 5 Department of Surgery and Science, Graduate School of Medical Sciences, Kyushu University, 3-1-1 Maidashi, Higashi-ku, Fukuoka 812-8582 Japan; 6 Kyushu Central Hospital of the Mutual Aid Association of Public School Teachers, 3-23-1 Shiobaru, Minami-ku, Fukuoka 815-8588 Japan

**Keywords:** Chemotherapy, DNA metabolism

## Abstract

Trifluridine (FTD), a tri-fluorinated thymidine analogue, is a key component of the oral antitumor drug FTD/TPI (also known as TAS-102), which is used to treat refractory metastatic colorectal cancer. Thymidine kinase 1 (TK1) is thought to be important for the incorporation of FTD into DNA, resulting in DNA dysfunction and cytotoxicity. However, it remains unknown whether TK1 is essential for FTD incorporation into DNA and whether this event is affected by the expression level of TK1 because *TK1*-specific-deficient human cancer cell lines have not been established. Here, we generated *TK1*-knock-out human colorectal cancer cells using the CRISPR/Cas9 genome editing system and validated the specificity of *TK1* knock-out by measuring expression of *AFMID*, which is encoded on the same locus as *TK1*. Using *TK1*-knock-out cells, we confirmed that TK1 is essential for cellular sensitivity to FTD. Furthermore, we demonstrated a correlation between the *TK1* expression level and cytotoxicity of FTD using cells with inducible *TK1* expression, which were generated from *TK1*-knock-out cells. Based on our finding that the *TK1* expression level correlates with sensitivity to FTD, we suggest that FTD/TPI might efficiently treat cancers with high *TK1* expression.

## Introduction

Trifluridine (FTD), a tri-fluorinated thymidine analogue, is a key component of the novel oral antitumor drug FTD/TPI (also known as TAS-102), which is used to treat refractory metastatic colorectal cancer based on an international phase III clinical trial^1^. The tri-phosphorylated form of FTD^2^ is thought to be incorporated into DNA through its replication during S phase^2–4^, resulting in DNA dysfunction and cytotoxicity.

Thymidine kinase 1 (encoded by *TK1*^5^) is a cytosolic nucleoside kinase that is part of the thymidine salvage pathway and mainly phosphorylates thymidine^6,7^. Based on its chemical structure, FTD is thought to be phosphorylated by TK1 and this modification is suggested to be essential for its cytotoxicity. Indeed, TK1 seems to be associated with the cytotoxicity of FTD^8,9^; however, the cell lines used in these previous reports were generated by random mutagenesis and not fully validated. Furthermore, although TK1-deficient cells play a key role in determining the importance of TK1 for FTD cytotoxicity, TK1-specific-deficient human cancer cell lines have not been established.

This study generated *TK1*-specific-knock-out human colorectal cancer cell lines and demonstrated that TK1 is essential for cellular sensitivity to FTD. Moreover, we provide the first evidence that the *TK1* expression level correlates with FTD sensitivity.

## Results

### *TK1* expression is indispensable for FTD cytotoxicity

**Figure 1:**
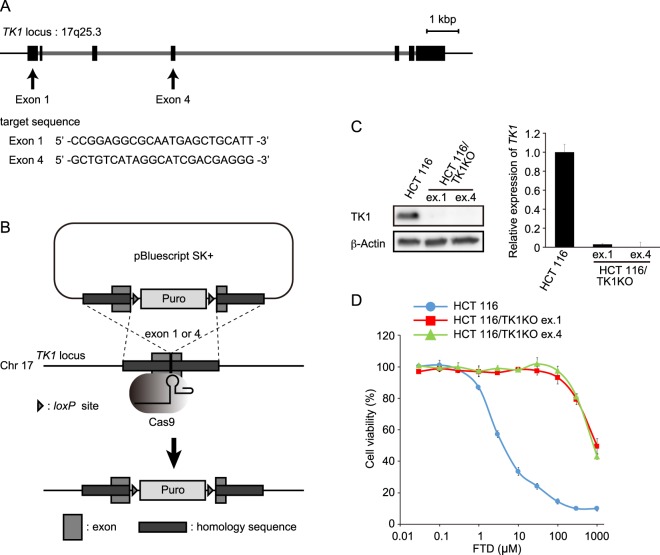
FTD cytotoxicity in *TK1*-knock-out cells. (**A**) Schematic diagram of the *TK1* locus on Chr17q25.3. Exons are denoted by black rectangles and introns are shown in light grey. (**B**) Experimental scheme of *TK1* knock-out. Three PCR fragments, 600–700 base pairs of the right and left homology arms and a puromycin resistance cassette, were cloned into pBluescript SK+. (**C**) Western blot analysis of TK1 protein (left) and quantitative RT-PCR analysis of *TK1* mRNA (right) in HCT 116 parental and *TK1*-knock-out cells. Expression of *TK1* was normalised against that of β-actin and is plotted relative to that in HCT 116 parental cells. Data are means ± s.d. of three independent experiments. (**D**) Cell viability assay. Cells were treated with nine points of dilution series of FTD for 3 days and then their viability was determined. The viability of cells not treated with FTD was defined as 100%. Data are means ± s.d. of three independent experiments.

We first examined the relationship between TK1 expression and FTD sensitivity in a panel of colorectal cancer cell lines. The TK1 expression level varied among the cell lines; however, there was no correlation between TK1 expression and FTD sensitivity (Fig. S1A,B). To exclude the possibility that differences in the genetic background among these cell lines influenced the results, we knocked down *TK1* to validate its importance for FTD cytotoxicity. Although *TK1*-knock-down cells tended to have a reduced sensitivity to FTD, it was not markedly different from that of control cells (Fig. S1C–F). To overcome this, we generated *TK1*-specific-knock-out HCT 116 human colorectal cancer cells using the CRISPR/Cas9 genome editing system. We used a knock-in strategy to generate these cells. Specifically, the *TK1* gene was targeted at two sites in exons 1 and 4, respectively (Fig. 1A), and puromycin resistance gene cassettes were integrated into the genome via homologous recombination (Fig. 1B). We obtained two puromycin-resistant HCT 116 cell lines; exon 1 was targeted in HCT 116/TK1KO ex.1 cells and exon 4 was targeted in HCT 116/TK1KO ex.4 cells. TK1 protein expression was completely abolished in both cell lines (Fig. 1C). To evaluate FTD sensitivity, HCT 116 parental and *TK1*-knock-out cells were treated with a dilution series of FTD comprising nine concentrations for 3 days and then their viability was determined. The HCT 116/TK1KO cell lines were more than 100-fold less sensitive to FTD than HCT 116 parental cells (Fig. 1D, Supplementary Table 1). The growth curve, doubling time (Fig. S2A), and cell cycle distribution (Fig. S2B) of HCT 116/TK1KO cells were similar to those of HCT 116 parental cells; therefore, the difference in FTD sensitivity between these two cell lines was due to their disparate *TK1* expression levels. *TK1*-knock-out RKO cells were also resistant to FTD (Fig. S3). Furthermore, a recent study reported a similar finding in *TK1*-knock-out DLD-1 cells^10^, suggesting that TK1 is essential for FTD cytotoxicity in a range of cell lines.

### *TK1* knock-out does not affect *AFMID* expression

**Figure 2:**
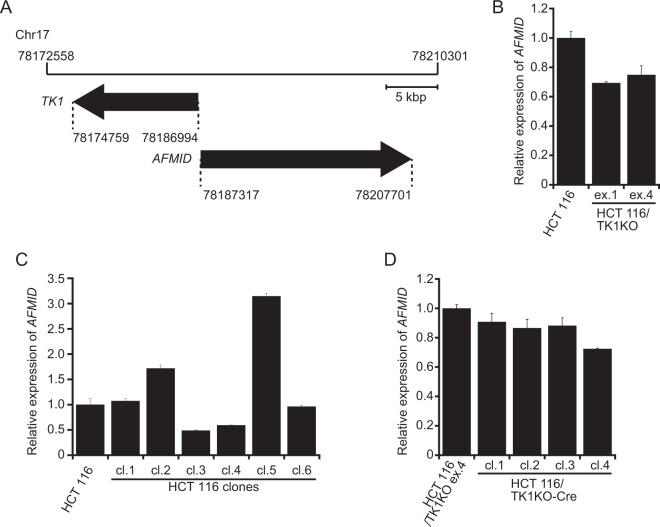
*AFMID* expression in *TK1*-knock-out cells. (**A**) Schematic diagram around the *TK1* locus on Chr17. (**B**–**D**) Expression of *AFMID* was determined by quantitative RT-PCR, normalised against that of *β-actin* and plotted relative to that in HCT 116 cells. (**B**) *TK1*-knock-out cell lines. (**C**) Cloned HCT 116 cell lines. (**D**) The HCT 116/TK1KO ex.4 cell line and its clones whose puromycin resistance cassettes were removed by the Cre-*loxP* recombination system. Data are means ± s.d. of three independent experiments.

*TK1* and *AFMID*, which encodes arylformamidase (Afmid, also known as kynurenine formamidase), are located on the same locus (*17q25*.*3*) and transcribed in opposite directions (Fig. 2A). Hence, knock-out of *TK1* might affect expression of *AFMID*. Indeed, although *TK1*-knock-out mice have been reported and exhibit kidney failure^11^, they do not express any native *Afmid* mRNA, and Afmid-specific activity is reduced to 0.1% in the liver^12^. Concurrent knock-out of two genes makes it difficult to determine which is responsible for the phenotype. Therefore, it was important to ensure that *TK1* was knocked out without affecting expression of *AFMID*. We first analysed *AFMID* expression in the *TK1*-knock-out cell lines. Expression of *AFMID* was approximately 30% lower in HCT 116/TK1KO ex.1 and HCT 116/TK1KO ex.4 cells than in HCT 116 parental cells (Fig. 2B). We hypothesised that *AFMID* expression may vary between individual cells of the HCT 116 parental line and that we selected clones with lower *AFMID* expression when generating the *TK1*-knock-out cell lines. To test this hypothesis, we isolated individual HCT 116 cells from the parental line by limiting dilution and analysed *AFMID* expression in each clone. The *AFMID* expression level varied between these clones (Fig. 2C), indicating that the expression level of *AFMID* in HCT 116 parental cells is an average of that in each individual cell. Alternatively, insertion of the puromycin resistance cassettes may have affected *AFMID* expression. To exclude this possibility, we investigated whether the *AFMID* expression level changed after removing the puromycin resistance cassettes from the *TK1* loci by Cre-*loxP* recombination. Removal of these cassettes did not affect *AFMID* expression (Fig. 2D). Therefore, the reduced *AFMID* expression in the *TK1*-knock-out cell lines was due to clonal variation, not to insertion of the puromycin resistance cassettes into the *TK1* loci. Thus, we conclude that *TK1*-specific-knock-out cell lines can be generated without affecting expression of *AFMID*.

### The *TK1* expression level correlates with FTD incorporation and cytotoxicity

**Figure 3:**
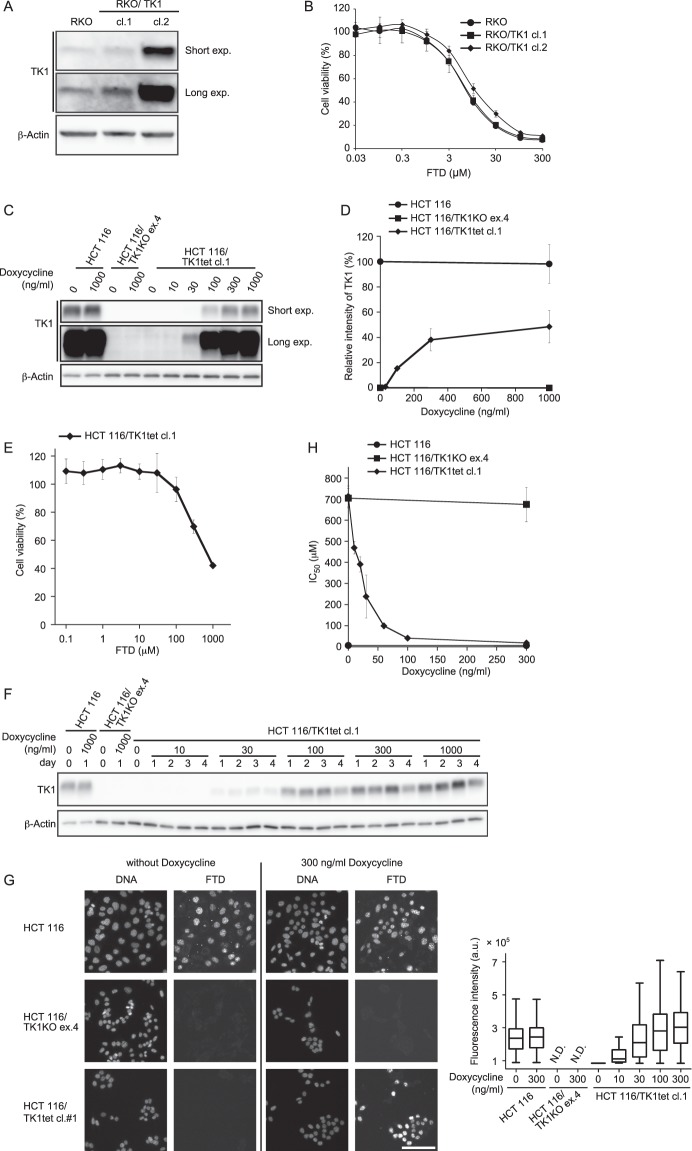
Correlation between the *TK1* expression level and FTD incorporation or cytotoxicity. (**A**,**B**) Lysates of RKO cells stably expressing *TK1* were immunoblotted with the indicated antibodies (**A**). Cells were treated with a dilution series of FTD comprising nine concentrations for 3 days and then their viability was determined. The viability of cells not treated with FTD was defined as 100%. Data are means ± s.d. of three independent experiments (**B**). (**C**,**D**) Western blot analysis (**C**) and quantification (**D**) of TK1 protein. Cells were treated with the indicated concentration of doxycycline for 1 day and the TK1 protein level was analysed. The intensity of the TK1 band was normalised against that of the β-actin band. The relative intensity of the TK1 band was calculated by setting that in cells not treated with doxycycline to 100%. Data are means ± s.d. of three independent experiments. (**E**) FTD sensitivity of cells with inducible *TK1* expression. HCT 116/TK1tet cl. 1 cells were treated with the indicated concentration of FTD for 3 days and then their viability was evaluated. Relative cell viability was calculated by setting that of cells not treated with FTD to 100%. Data are means ± s.d. of three independent experiments. (**F**) Western blot analysis of TK1. Cells were treated with the indicated concentration of doxycycline for the indicated number of days. (**G**) Immunofluorescence images of FTD-incorporated cells (left). Cells were treated with the indicated concentration of doxycycline for 1 day and then with 6.4 μM FTD for 1 h, fixed and immunostained with an anti-BrdU antibody. Fluorescence intensities of FTD incorporated into genomic DNA were quantified (right). Scale bar, 100 μm. (**H**) FTD sensitivity of cells. HCT 116 parental cells, *TK1*-knock-out cells and cells with inducible *TK1* expression were treated with the indicated concentration of doxycycline for 1 day and then with nine points of dilution series of FTD for 3 days. The IC_50_ values in each cell line at the indicated concentration of doxycycline were calculated and plotted. Data are means ± s.d. of three independent experiments.

The relationship between *TK1* knock-out and cytotoxicity of FTD was described above (Fig. 1); however, it was unknown whether FTD cytotoxicity correlates with the expression level of *TK1*. To address this question, we first examined FTD cytotoxicity in *TK1*-overexpressing RKO cells, in which basal expression of TK1 was low (Fig. S1A). *TK1*-overexpressing RKO cells did not exhibit increased sensitivity to FTD, suggesting that an excess amount of TK1 protein does not enhance FTD sensitivity (Fig. 3A,B). We next investigated whether the *TK1* expression level correlates with FTD cytotoxicity when expression of *TK1* is lower than the endogenous level. To this end, we generated cells with inducible *TK1* expression (hereafter referred to as HCT 116/TK1tet and RKO/TK1tet cells), in which *TK1* expression was induced by doxycycline treatment, using *TK1*-knock-out cells. In these cells, TK1 expression was efficiently induced by treatment with doxycycline for 1 day, and the expression level correlated well with the concentration of doxycycline (Figs 3C,D and S4A). The expression level of TK1 plateaued at 1000 ng/ml doxycycline and was about one-half of that in HCT 116 parental cells. Without doxycycline treatment, HCT 116/TK1tet cell lines were resistant to FTD, similar to *TK1*-knock-out cells (Figs 3E and S4B). Furthermore, the effect of doxycycline on TK1 expression persisted for 4 days (Figs 3F and S4C), meaning TK1 expression was sustained throughout all experiments. HCT 116/TK1tet cell lines grew slightly slower than HCT 116/TK1KO ex.4 and HCT 116 parental cells (Fig. S2A). This might be caused by stable integration of the *TK1* expression-inducible plasmids. Doxycycline did not affect the growth (Fig. S2A) or cell cycle distribution (Fig. S2B) of any cell line *per se*.

To evaluate whether FTD was incorporated into genomic DNA of HCT 116/TK1KO ex.4 or HCT 116/TK1tet cell lines, we treated these cells with 6.4 μM FTD, which was the half maximal inhibitory concentration (IC_50_) when HCT 116 parental cells were treated with FTD for 3 days without doxycycline (Fig. 1D and Supplementary Table 1), for 1 h and analysed the amount of incorporated FTD. Consistent with our previous report^13^, FTD was rapidly incorporated into nuclear DNA in HCT 116 parental cells, whereas it was hardly detectable in *TK1*-knock-out cells, and these results were not affected by addition of doxycycline (Fig. 3G). In HCT 116/TK1tet cell lines, the amount of incorporated FTD correlated with the doxycycline concentration (Figs 3G, S4D and Supplementary Table 2). These data indicate that the amount of FTD incorporated into nuclear DNA depends on the *TK1* expression level. As described above, *TK1*-expressing cells were sensitive to FTD, whereas *TK1*-knock-out cells were highly resistant to FTD (Figs 1D, 3B and S4B). Next, we investigated whether FTD cytotoxicity is dependent on the *TK1* expression level. To compare FTD sensitivity between cell lines, we calculated IC_50_ values in the presence of various concentrations of doxycycline. While doxycycline did not affect FTD cytotoxicity in HCT 116 parental or *TK1*-knock-out cells, FTD sensitivity correlated well with the doxycycline concentration in HCT 116/TK1tet cell lines (Figs 3H, S4E and Supplementary Table 1). FTD sensitivity also correlated with the doxycycline concentration in RKO/TK1tet cell lines (Fig. S4F,G). These results indicate that the *TK1* expression level highly correlates with cellular sensitivity to FTD in an isogenic background.

## Discussion

Previous reports indicate that TK1 is indispensable for FTD cytotoxicity^8,9^; however, the cells used were not certified to be specifically *TK1*-deficient. In this study, for the first time, we generated *TK1*-specific-knock-out human cancer cell lines using the CRISPR/Cas9 genome editing system and then obtained cell lines with inducible *TK1* expression. Using these cells, we confirmed that *TK1* was essential for cellular sensitivity to FTD. Furthermore, we demonstrated that the *TK1* expression level correlated with FTD sensitivity.

Although *TK1*-knock-out mice have been reported, they are also deficient in *Afmid*, a formamidase that is part of the tryptophan catabolism pathway^11,12^. *TK1* and *AFMID* are located on the same locus; therefore, knock-out of *TK1* may affect expression of *AFMID*. Simultaneous knock-out of two genes hinders elucidation of the function of each gene. Considering the potential influence on *AFMID* expression, we targeted exon 4 of *TK1* to generate *TK1*-knock-out cells. These cells did not exhibit changes in growth, cell cycle distribution, or *AFMID* expression. Thus, we conclude that the phenotypes of *TK1*-knock-out cells were due to their lack of *TK1* expression. In comparison with HCT 116 parental cells, *TK1*-knock-out cells were highly resistant to FTD. This demonstrated that *TK1* expression was indispensable for FTD to exert cytotoxicity and excluded the possibility that AFMID was involved in FTD cytotoxicity.

We found no correlation between TK1 expression and FTD sensitivity in a panel of colorectal cancer cell lines. Furthermore, overexpression of TK1 did not increase the sensitivity of cells to FTD. Other factors, in addition to the TK1 expression level, might determine FTD sensitivity. Indeed, nucleoside transporters (hENT1, hENT2 and hCNT1)^13–16^ contribute to FTD cytotoxicity. Furthermore, nucleoside kinases, such as TMPK and NDK, are predicted to be involved in FTD phosphorylation. Hence, overexpression of TK1 alone is insufficient to increase the sensitivity of cells to FTD. On the other hand, doxycycline-inducible expression of *TK1* increased FTD sensitivity in a doxycycline dose-dependent manner in cells with the same genetic background as *TK1*-knock-out cells. These results strongly indicate that the *TK1* expression level highly correlates with cellular cytotoxicity to FTD in an isogenic background because the *TK1* expression level was dependent on the doxycycline concentration and doxycycline did not affect cell growth or the cell cycle. Together, these data suggest that FTD would more effectively treat cancers with high *TK1* expression than those with low *TK1* expression.

It was reported that thymidine kinase activity is higher in the tumour than in normal tissue from the same patient^17^. Furthermore, several studies report that high expression of *TK1* significantly correlates with poor prognosis in various types of cancer, and the *TK1* expression level is thus considered to be a prognostic factor^18–22^. Cancers that highly express *TK1* may be efficiently treated with FTD/TPI. Indeed, recent studies discussed the importance of the TK1 expression level as a predictive factor of FTD/TPI efficacy in metastatic colorectal cancer patients^23,24^. Future studies should determine the expression levels of *TK1* in clinical specimens of various types of cancer. These findings are expected to help determine which types of cancer are particularly susceptible to FTD/TPI treatment.

## Methods

### Cell culture and reagents

HCT 116 (ECACC; 91091005), HCT 116 (ATCC; CCL-247), HT-29 (ATCC; HTB-38), LoVo (ATCC; CCL-229), LS1034 (ATCC; CRL-2158), LS411N (ATCC; CRL-2159), RKO (ATCC; CRL-2577), SW48 (ATCC; CCL-231) and SW480 (ATCC; CCL-228) cells were cultured in high-glucose Dulbecco’s modified Eagle’s medium containing 4 mM L-glutamine and 1 mM sodium pyruvate (Thermo Fisher Scientific) and supplemented with 10% Tet-tested foetal bovine serum (Thermo Fisher Scientific), 100 U/ml penicillin and 100 μg/ml streptomycin (Nacalai Tesque) at 37 °C in 5% CO_2_. FTD was purchased from Tokyo Chemical Industry. Doxycycline was purchased from Takara-Clontech. All reagents were solubilized in distilled water. *TK1*-specific siRNA was synthesized by Thermo Fisher Scientific and the sequence is provided in Supplementary Table 3.

In RNAi experiments, Stealth RNAi Negative Control Med GC (Thermo Fisher Scientific) was used as a control and siRNA was transfected using Lipofectamine RNAiMAX (Thermo Fisher Scientific).

### Plasmid construction

To knock-out *TK1* using the CRISPR/ Cas9 genome editing system, guide RNA (gRNA) sequences were designed using the online software CRISPRdirect^25^. The targeting sequences of gRNAs are provided in Fig. 1A and Supplementary Table 3. The sense and antisense oligonucleotides were annealed and cloned into the *Bbs*I site of pX330-U6-Chimeric_BB-CBh-hSpCas9 (Addgene plasmid #42230), which was a gift from Feng Zhang^26^. To construct donor vectors by PCR, template DNA (genomic DNA of HCT 116 cells) was amplified using KOD FX DNA polymerase (TOYOBO) and primers containing a sequence homologous to the target locus, which are provided in Supplementary Table 3. The amplified left- and right-arm DNA fragments, which contained approximately 600–700 base pairs homologous to the target locus, and a puromycin resistance cassette were cloned into the *BamH*I–*Not*I site of pBluescript SK+ using an In-Fusion HD Cloning Kit (Takara-Clontech). To construct the stable and inducible *TK1* expression plasmids, the *TK1* gene was amplified from template DNA (total cDNA of HCT 116 cells) using KOD FX DNA polymerase (TOYOBO) and primers (Supplementary Table 3), and then cloned into the pcDNA3.1 (Thermo Fisher Scientific) and pTetOne (Takara-Clontech) vectors using an In-Fusion HD Cloning Kit (Takara-Clontech), respectively.

### Generation of *TK1*-knock-out cell lines and cell lines with stable and inducible *TK1* expression

To establish *TK1*-knock-out cells, HCT 116 and RKO cells were co-transfected with a CRISPR-Cas9 vector and a donor vector. Forty-eight hours later, cells were selected with 500 ng/ml puromycin (Thermo Fisher Scientific). The puromycin resistance cassettes integrated into the *TK1* loci were removed by the Cre-*loxP* recombination system (Fig. 2B). Cre recombinase proteins were delivered into *TK1*-knock-out cells using Cre Recombinase Gesicles (Takara-Clontech). To establish cells with stable *TK1* expression, RKO cells were transfected with the pcDNA3.1-TK1 plasmid. Forty-eight hours later, cells were selected with 500 μg/ml G418 (Geneticin; Thermo Fisher Scientific). To establish cells with inducible *TK1* expression, *TK1*-knock-out HCT 116 and RKO cells were co-transfected with a 50:1 ratio of pTetOne-TK1:linear hygromycin marker, according to the manufacturer’s protocol (Takara-Clontech). Forty-eight hours later, cells were selected with 400 μg/ml hygromycin B (Thermo Fisher Scientific). All transfections were carried out using the 4D Nucleofector system (Lonza).

### Western blotting

Cells were lysed in RIPA buffer [50 mM Tris-HCl (pH 8.0), 150 mM NaCl, 1.0% Nonidet P-40, 0.5% sodium deoxycholate and 0.1% sodium dodecyl sulfate] containing 1 mM PMSF and appropriate concentrations of a protease inhibitor cocktail (Nacalai Tesque) and a phosphatase inhibitor cocktail (Nacalai Tesque). Cell extracts were clarified by centrifugation. The supernatant was boiled in SDS sample buffer (Nacalai Tesque). An ImageQuant LAS-4000 mini system (GE Healthcare) was used to detect chemiluminescence. Signal intensities were quantified using ImageQuant TL software (GE Healthcare). The following antibodies were used at the indicated dilutions: anti-TK1 (1:5000; clone EPR3193, Abcam) and anti-β-actin (1:10,000; clone AC-74, Sigma). Images were cropped for presentation. Uncropped immunoblots are shown in Supplementary Fig. 5.

### Quantitative reverse-transcription PCR (RT-PCR)

Total RNA was extracted from each cell line using an RNeasy Mini Kit (Qiagen). cDNA was synthesized with a High-Capacity cDNA Reverse Transcription Kit (Applied Biosystems) using the primers provided in Supplementary Table 3. mRNA expression was normalised against that of *β-actin*. Quantitative RT-PCR was performed with a QuantiFast SYBRGreen PCR Kit (Qiagen). Fluorescence signals were detected by a LightCycler 480 system (Roche Diagnostics).

### Cell viability assay

Cell viability was evaluated by the CellTiter-Glo 2.0 Assay (Promega) according to the manufacturer’s protocol. Briefly, cells were plated in a 96-well plate at a density of 500 cells per well in 100 μl of growth medium. The next day, 50 μl of growth medium containing 3x final concentration of doxycycline was added to each well. One day later, 50 μl of medium containing 4x final concentration of FTD and the final concentration of doxycycline was added. Three days later, 100 μl of culture medium was removed, 100 μl of CellTiter-Glo 2.0 Assay reagent was added and the sample was agitated to uniformly lyse cells. Luminescence was detected by a TriStar LB941 reader (Berthold Technologies). The ratio of the luminescence of each sample to that of the mock-treated sample was determined, and the IC_50_ value was calculated with XLfit (IDBS). The ‘dose response 205′ program was used for curve fitting. Each experiment was repeated three times. To determine the cell growth rate, the RealTime-Glo MT Cell Viability assay (Promega) was performed according to the manufacturer’s protocol. Briefly, cells were treated with an appropriate concentration of MT Cell Viability Substrate and NanoLuc Enzyme. Luminescence was detected by a TriStar LB941 reader at 1 h and various other time points after cell plating.

### Detection of FTD incorporated into nuclear DNA

FTD was detected as described in our previous report^13^. Briefly, cell seeding and addition of doxycycline and FTD were performed as described for the cell viability assay. One hour after FTD addition, cells were fixed in ice-cold 70% ethanol for 5 min and treated with 1.5N HCl for 1 h. After blocking with goat serum, FTD was detected with an anti-BrdU antibody (1:250; clone 3D4; BD Biosciences). Samples were treated with an Alexa Fluor 488-conjugated goat anti-mouse IgG secondary antibody (1:200; Thermo Fisher Scientific) and 1 μg/ml 4′, 6-diamidino-2-phenylindole, dihydrochloride (DOJINDO). Fluorescence was detected with a Cytell Cell Imaging System (GE Healthcare). Images were analysed using IN Cell Analyzer Workstation 3.7.1. The maximum fluorescence intensity in cells cultured without FTD was determined as the background. Outliers and signals less than the background were excluded. To calculate outliers, the upper quartile (Q_3/4_) and lower quartile (Q_1/4_) were first determined. Then, the interquartile range (IQR) was calculated as Q_3/4_–Q_1/4_. Data that were more than Q_3/4_ + 1.5 × IQR or less than Q_1/4_ − 1.5 × IQR were defined as outliers.

### Cell cycle analysis

Cells were harvested by trypsinization after 1 day of doxycycline treatment, fixed with 70% ethanol and stained with propidium iodide. Fluorescence was analysed using a FACSCalibur instrument (BD Biosciences). The cell cycle was evaluated by DNA content analysis.

## Supplementary information

Suppl info
