# Supplementary material for: Cytotoxicity of trifluridine correlates with the thymidine kinase 1 expression level
Source: Sci Rep. 2019 May 28;9:7964. doi: 10.1038/s41598-019-44399-6 (PMC6538667; doi:10.1038/s41598-019-44399-6)
Supplement: Supplementary file 1 — Suppl info [file 41598_2019_44399_MOESM1_ESM.pdf]

Supplementary information for

Cytotoxicity of trifluridine correlates with the thymidine kinase 1 expression level

Yuki Kataoka[1,2], Makoto Iimori[1*], Shinichiro Niimi[3], Hiroshi Tsukihara[4], Takeshi Wakasa[1,4], Hiroshi Saeki[5], Eiji Oki[5], Yoshihiko Maehara[5,6] and Hiroyuki Kitao[1,3]

1: Department of Molecular Cancer Biology, Graduate School of Pharmaceutical Sciences,Kyushu University,
3-1-1 Maidashi, Higashi-ku, Fukuoka, 812-8582, Japan.

2: Drug Discovery & Development I Laboratory, Taiho Pharmaceutical Co. Ltd., Japan.
3, Okubo, Tsukuba, Ibaraki, 300-2611, Japan.

3: Innovative Anticancer Strategy for Therapeutics and Diagnosis Group, Innovative Center for Medical Redox Navigation, Kyushu University, Japan.
3-1-1 Maidashi, Higashi-ku, Fukuoka, 812-8582, Japan.

4: Translational Research Laboratory, Taiho Pharmaceutical Co., Ltd.,
224-2, Ebisuno Hiraishi, Kawauchi-Cho, Tokushima, 771-0194, Japan.

5: Department of Surgery and Science, Graduate School of Medical Sciences, Kyushu University, Japan.
3-1-1 Maidashi, Higashi-ku, Fukuoka, 812-8582, Japan.

6: Kyushu Central Hospital of the Mutual Aid Association of Public School Teachers, 3-23-1 Shiobaru, Minami-ku, Fukuoka, 815-8588, Japan.

*Corresponding author: Makoto Iimori
Phone/FAX: +81-92-642-6499; E-mail: iimori@surg2.med.kyushu-u.ac.jp

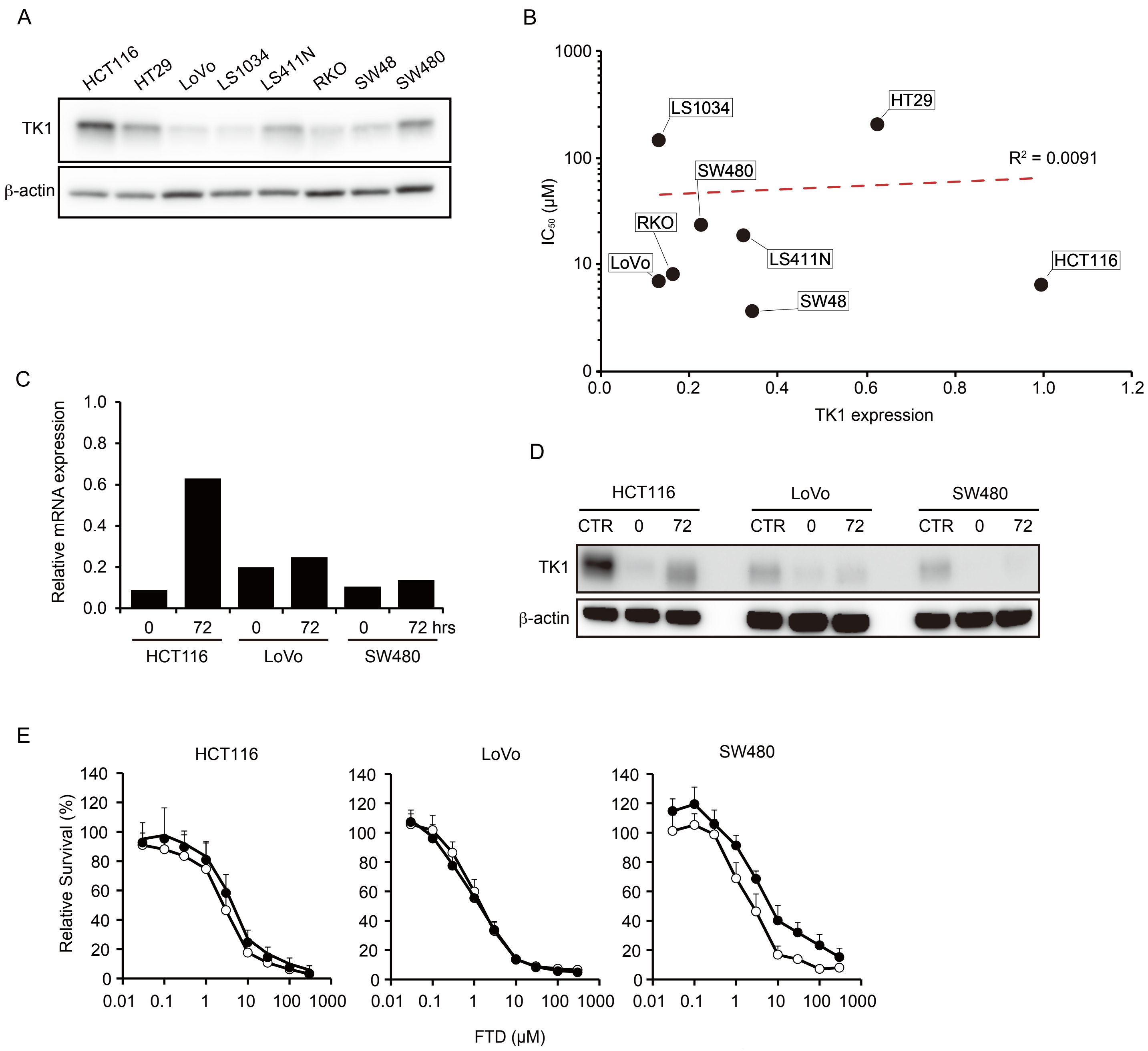

F

	IC50 (μM)		
	siControl	siTK1	fold change
HCT116	2.6 ± 1.6	4.2 ± 1.3	1.6
LoVo	1.6 ± 0.3	1.4 ± 0.2	0.9
SW480	2.6 ± 0.7	10 ± 3.4*	4.0

Supplementary figure 1. FTD cytotoxicity in TK1-knock-down colorectal cancer cells.
(**A**,**B**) Colorectal cancer cell lines were immunoblotted with the indicated antibody. Correlation analysis between FTD cytotoxicity and the TK1 expression level in A. (**C**,**D**) Cells were transfected with *TK1*-targeting siRNA. At 48 and 120 h after transfection, which are indicated as 0 and 72 h, respectively, cells were harvested and the mRNA (**C**) and protein (**D**) levels of TK1 were measured. (**E**) Influence of *TK1* knock-down on cytotoxicity of FTD. At 48 h after transfection with control (open circles) or *TK1*-targeting (closed circles) siRNA, cells were treated with FTD for 72 h and their viability was determined. All data are expressed as means ± s.d. (**F**) Summary of the IC50 values in each cell line in (**E**).

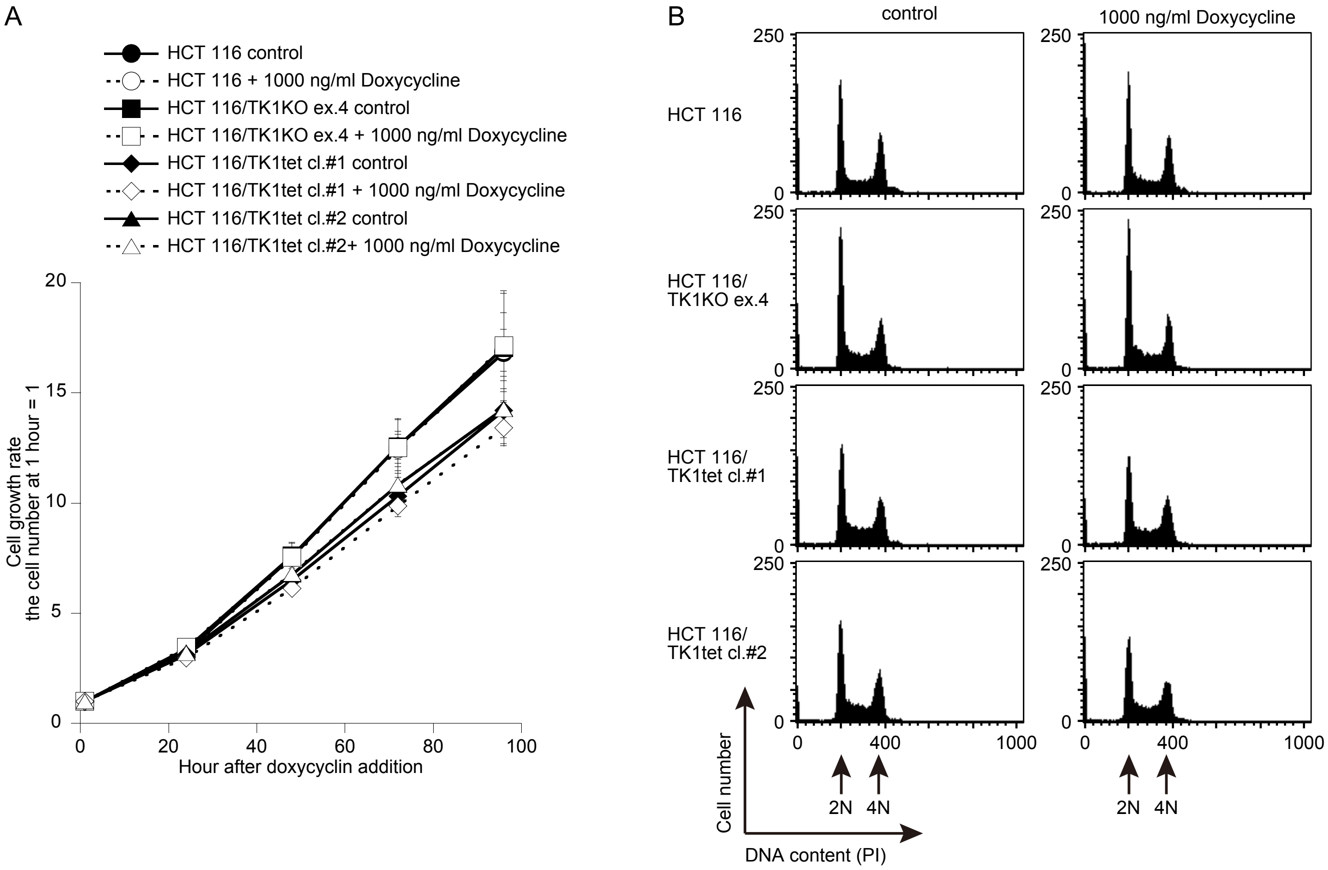

Supplementary figure 2. Cell growth rates and cell cycle distributions.

(**A**) Growth curve of each cell line treated with 0 or 1000 ng/ml doxycycline. Cell viability was evaluated at 1, 24, 48, 72 and 96 h after plating, and the cell growth rate relative to that at 1 h was calculated. (**B**) Cell cycle distribution. Cells were treated with 0 or 1000 ng/ml doxycycline for 1 day, fixed and stained with propidium iodide. The DNA content was quantitated using a FACSCalibur instrument.

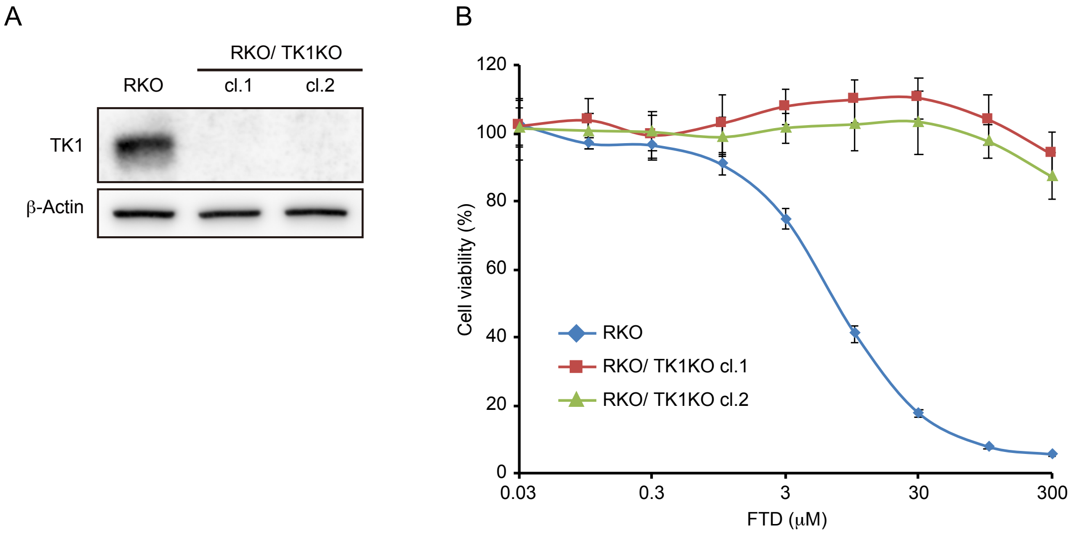

Supplementary figure 3. FTD cytotoxicity in *TK1*-knock-out RKO cells.

(**A**) Western blot analysis of TK1 proteinin RKO parental and *TK1*-knock-out cells. (**B**) Cell viability assay. Cells were treated with nine points of dilution series of FTD for 3 days and then their viability was determined. The viability of cells not treated with FTD was defined as 100%. Data are means ± s.d. of three independent experiments.

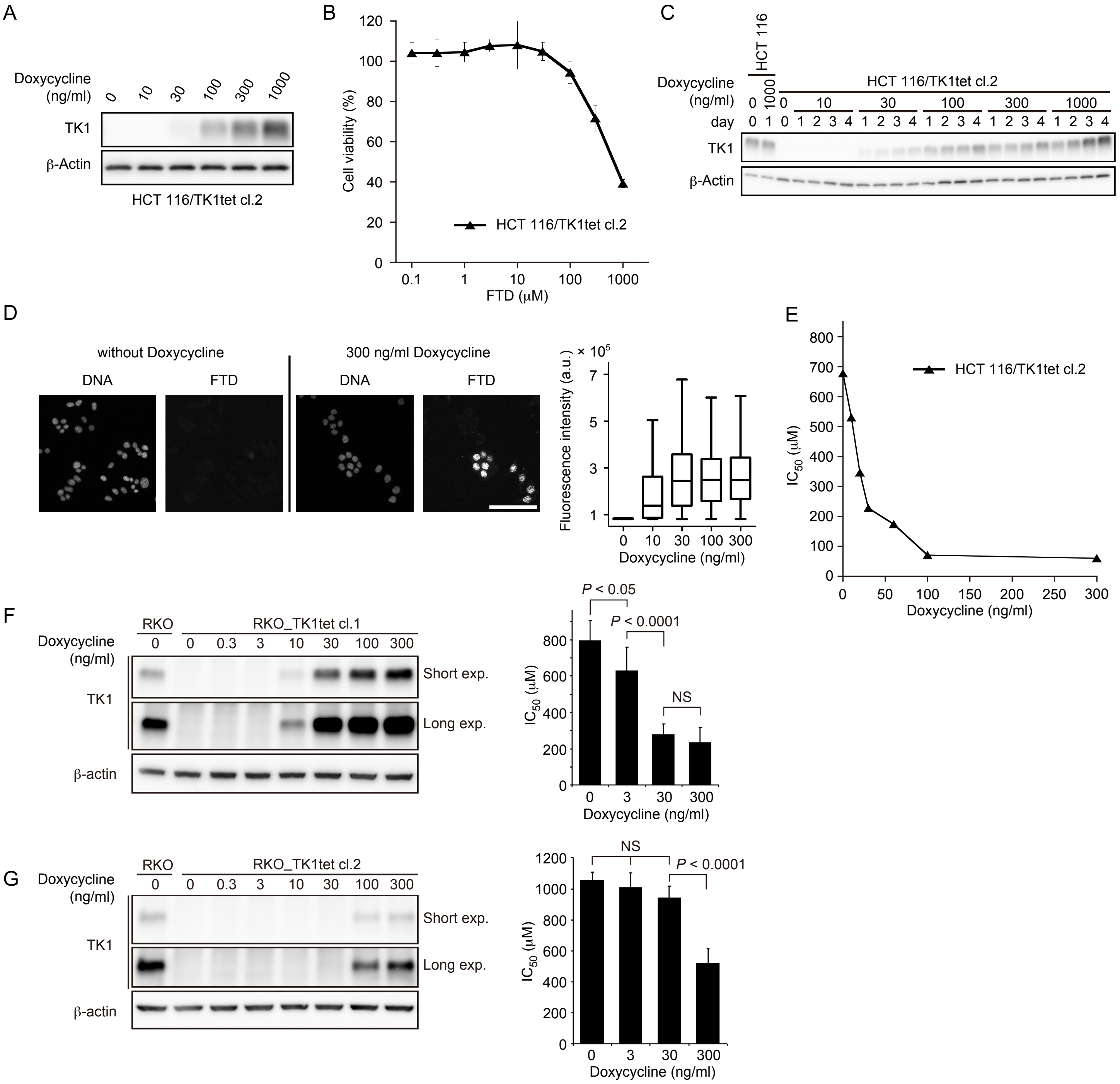

Supplementary figure 4. Response of HCT 116/TK1tet cl. 2 and RKO/TK1tet cells to doxycycline and FTD.

(**A**) Western blot analysis of TK1. HCT 116/TK1tet cl. 2 cells were treated with the indicated concentration of doxycycline for 1 day and then the level of TK1 was analysed. (B) Sensitivity of HCT 116/TK1tet cl. #2 cells to FTD. Cells were treated with the indicated concentration of FTD for 3 days and then their viability was evaluated. Relative cell viability was calculated by setting that of cells not treated with FTD to 100%. (**C**) Western blot analysis of TK1. Cells were treated with the indicated concentration of doxycycline for the indicated number of days. (**D**) Immunofluorescence images of FTD-incorporated cells (left). Cells were treated with the indicated concentration of doxycycline for 1 day and then with 6.4 µM FTD for 1 h, fixed and immunostained with an anti-BrdU antibody. Fluorescence intensities of FTD incorporated into genomic DNA were quantified (right). Scale bar, 100 µm. (**E**) FTD sensitivity of HCT 116/TK1tet cl. 2 cells. Cells were treated with the indicated concentration of doxycycline for 1 day and then with nine points of dilution series of FTD for 3 days. The IC50 values in HCT 116/TK1tet cl. #2 cells treated with the indicated concentrations of doxycycline were calculated and plotted. (**F, G**) Western blot analysis of TK1(left) and FTD sensitivity (right) . RKO/TK1tet cells were treated with the indicated concentration of doxycycline for 1 day and then the level of TK1 and IC50 values were analysed. Data are means± s.d. from six independent experiments. Two-tailed t-test, NS, not significant.

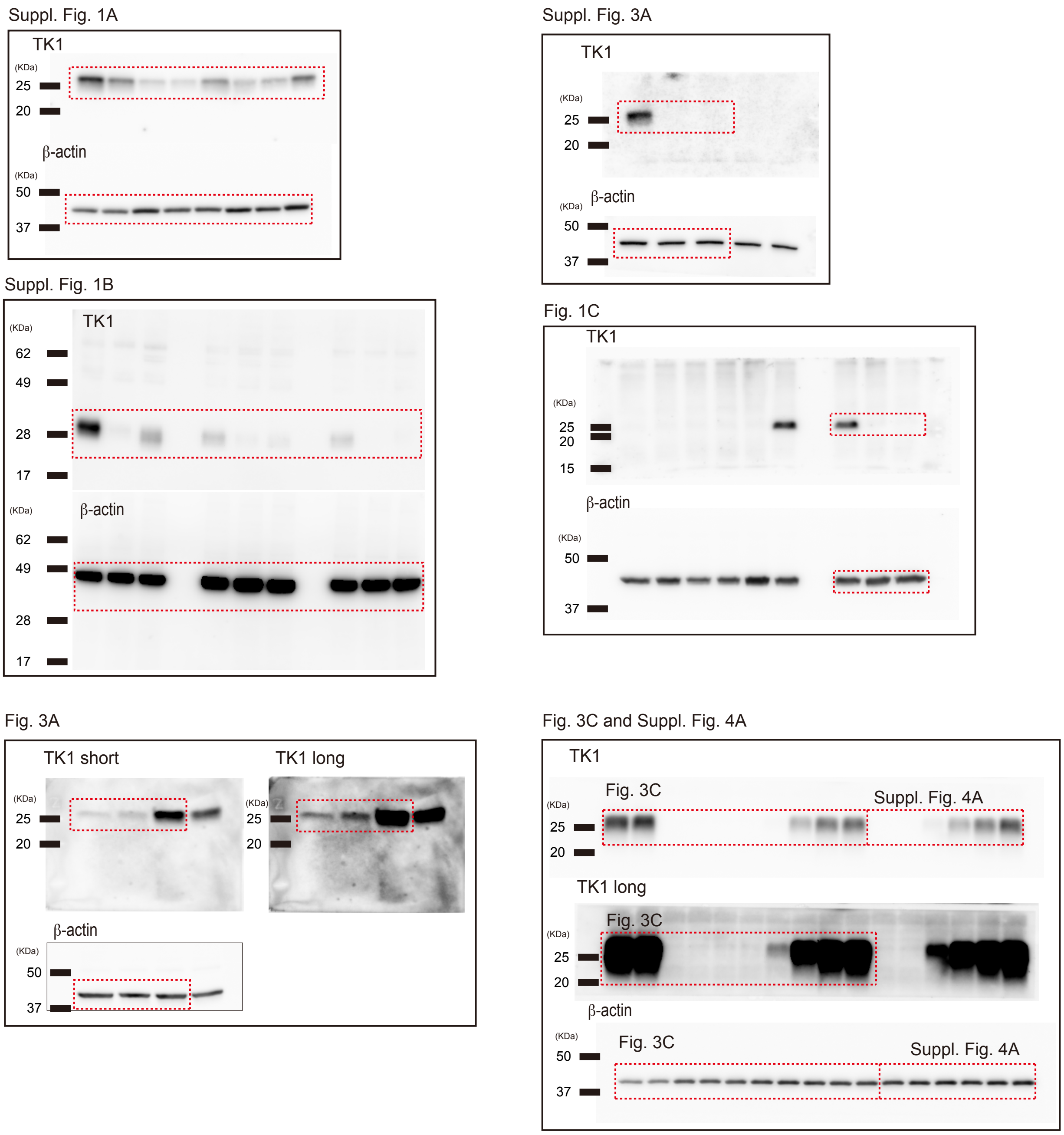

Supplementary figure 5. Uncropped images of immunoblot. Red boxes show cropped regions.

Fig. 3F and Suppl. Fig. 4C

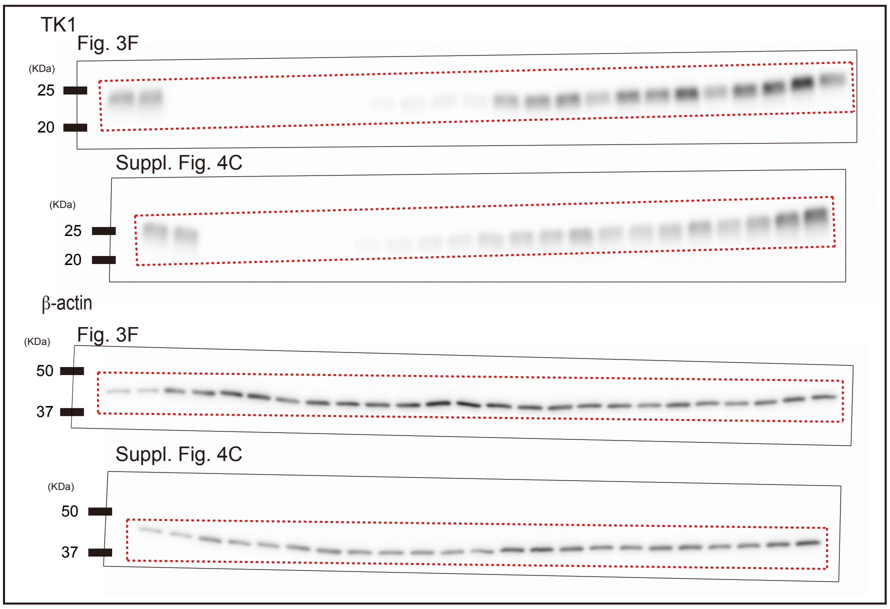

Suppl. Fig. 4F,G

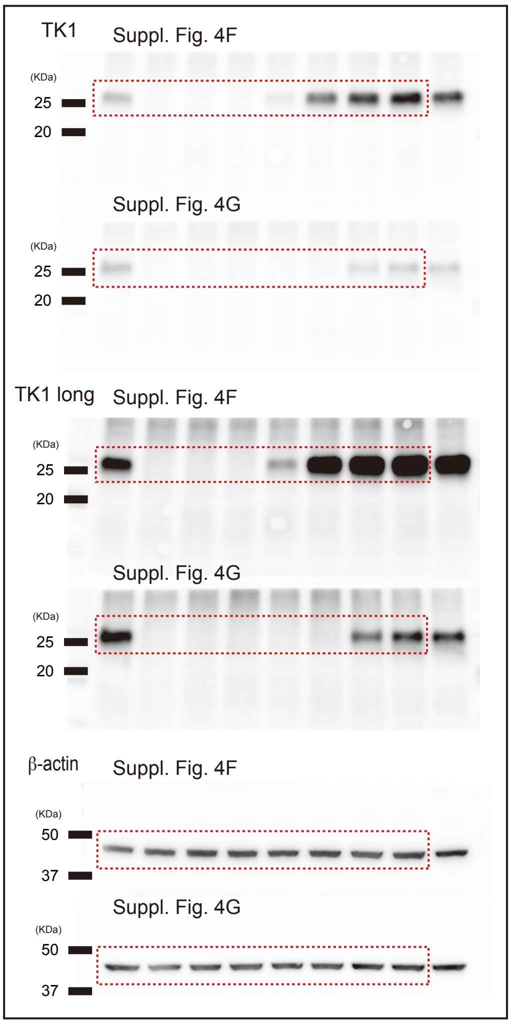

Supplementary figure 5. Continued

Supplemental Table 1. IC_{50} value of FTD in each cell line

FTD IC_{50} (Average ± SD, μM)

	Doxycycline (ng/ml)						
Cell line	0	10	20	30	60	100	300
HCT 116	6.4 ± 1.6	-	-	-	-	-	6.0 ± 1.3
HCT 116/TK1KO ex.4	710 ± 47	-	-	-	-	-	670 ± 81
HCT 116/TK1tet cl.1	710 ± 50	470 ± 30	390 ± 36	240 ± 100	99 ± 14	41 ± 4.6	18 ± 4.7
HCT 116/TK1tet cl.2	680 ± 150	530 ± 64	350 ± 42	230 ± 53	170 ± 40	71 ± 10	60 ± 18

Supplemental Table 2. Incorporation of FTD into DNA

Cell line	HCT 116			HCT 116/TK1KO ex.4			HCT 116/TK1tet cl.9						HCT 116/TK1tet cl.16					
FTD (μM)	0	6.4		0	6.4		0	6.4					0	6.4				
Dox (ng/ml)	0	0	300	0	0	300	0	0	10	30	100	300	0	0	10	30	100	300
all data	4262	5131	5817	3245	3995	3817	2611	2542	2414	2295	2431	2425	3432	3553	3515	3462	3346	2285
usable data	4258	2746	3239	3242	0	0	2604	4	32	197	812	1065	3411	8	81	474	1056	863
lower quartile	44769	171743	179664	42188	-	-	44013	85325	93386	124525	163856	206428	43176	82053	87248	138799	159392	168398
median	54079	235900	244067	51660	-	-	52835	85490	111675	210197	280886	303336	50885	83127	139099	245179	249001	247708
upper quartile	62630	293967	299302	60686	-	-	61065	85600	164557	317609	382829	391801	58651	83930	261412	357046	337297	344684
IQR	17861	122223	119638	18498	-	-	17051	275	71171	193084	218973	185373	15476	1877	174163	218247	177905	176286
minimum	19059	89408	89392	19775	-	-	20609	85075	86160	84814	84910	84867	20329	81493	81477	81431	81533	81628
maximum	89356	474389	472941	87281	-	-	84808	85682	243982	571356	708414	639776	81404	85250	504021	677535	600956	607390

Supplemental Table 3. Primer, gRNA and siRNA sequences

Primer	Sequence (5'-3')	Application
TK1_KO_exon1_sense	CACCGAATGCAGCTCATTGCGCCTC	gRNA for pX330
TK1_KO_exon1_antisense	AAACGAGGCGCAATGAGCTGCATTC	
TK1_KO_exon4_sense	CACCGGCTGTCATAGGCATCGACGA	
TK1_KO_exon4_antisense	AAACTCGTCGATGCCTATGACAGCC	
TK1_exon1_L-arm_F	GCAGCCCGGGGGATCCCTGGCAGGGTCTACGGATATTATTAGC	PCR to construct donor vectors
TK1_exon1_L-arm_R	TATACGAACGGTAGGAAGTTCACGAACCCGAGTACTCTCCAA	
TK1_exon1_R-arm_F	TATACGAACGGTAGGAGCTGCATTAACCTGCCCACTGT	
TK1_exon1_R-arm_R	CGCGGTGGCGGCCGCCCACGGCTTCAGACTCCTTGGTTT	
TK1_exon4_L-arm_F	GCAGCCCGGGGGATCCCAGCTCCTGAACAGTGGAAGAGTT	
TK1_exon4_L-arm_R	TATACGAACGGTAGGATGCCTATGACAGCCACGCCCAGG	
TK1_exon4_R-arm_F	TATACGAACGGTAGGAGTTTGTAAGTTGGCTTGTCTTGGCA	
TK1_exon4_R-arm_R	CGCGGTGGCGGCCGCTGTGCGCTGCTATGACTGGCTAATTTCT	
TK1_F	CCCTCGTAAAGAATTCACCATGAGCTGCATTAACCTGCCC	PCR of TK1 for insertion into pTetOne
TK1_R	GCAGAGATCTGGATCCTCAGTTGGCAGGGCTGCATTG	
TK1_F	GGCAGTTTTTCCCTGACATC	Quantitative RT-PCR
TK1_R	CCTCGACCTCCTTCTCTGTG	
AFMID_F	ACTGGGAGCAGAGGAAGCCTTGA	
AFMID_R	GACATGCAGCAGGCTCTTCCT-3	
β-actin_F	CTGGCACCACACCTTCTACAATG	
β-actin_R	GGCGTACAGGGATAGCACAGC	
TK1_siRNA	CUCGCUACAGCAGCAGCUUdTdT	RNAi

Supplementary methods

The experiments of supplemental figure 1 were performed as follows.

Cytotoxicity test

siRNA-transfected cells were seeded at densities of 1×10^3 (HCT116 cells) or 3×10^3 (LoVo and SW480 cells) cells/well in 96-well plates, incubated for 24 h, and then treated with FTD for 72 h. Cell numbers were determined using a simplified crystal violet staining method.

Quantitative real-time reverse transcription polymerase chain reaction (RT-qPCR)

Cells were replated 48 hours after siRNA transfection. FTD was added at 24 hours after replating. mRNA expression was quantified at 0 and 72 hours after FTD addition. RT-qPCR was performed on a PRISM 7900HT sequence detection system using TaqMan Universal PCR Master Mix (Applied Biosystems) with the following amplifications conditions: 50°C for 2 min, 95°C for 10 min, 40 cycles of 95°C for 15 s, and 60°C for 1 min. The expression level of *TK1* was normalized to those of β-actin (*ACTB*). The primers and TaqMan probes were prepared using Assay-on-Demand gene-expression products (Applied Biosystems). Probe ID was Hs01062123_m1 for *TK1*. The human *ACTB* probe (VIC/MGB Probe; Applied Biosystems) was used as an endogenous control.
